# An Example of a Novel Efficient Plant Extraction Technique: Electromagnetic Induction Heating

**DOI:** 10.3390/molecules23113048

**Published:** 2018-11-21

**Authors:** Francesco Epifano, Francesca Preziuso, Vito Alessandro Taddeo, Serena Fiorito, Salvatore Genovese

**Affiliations:** Dipartimento di Farmacia, Università “G. d’Annunzio” of Chieti-Pescara, Via dei Vestini 31, 66100 Chieti Scalo (CH), Italy; francesca.preziuso@unich.it (F.P.); vito.taddeo@unich.it (V.A.T.); serena.fiorito@unich.it (S.F.); s.genovese@unich.it (S.G.)

**Keywords:** anthraquinone, electromagnetic induction heating, emodin, extraction methodologies, HPLC, *Rheum palmatum*

## Abstract

A simple and easy to handle extraction procedure based on the use of electromagnetic induction heating is described. To assess the potential, scopes, and limitations of this novel process, extraction and subsequent HPLC quantification of emodin from an hydroalcoholic extract of rhizome of *Rheum palmatum* (Chinese rhubarb) was selected as the reference experiment. Maceration at room temperature and by heating, ultrasound-assisted, and microwave-assisted extractions were also carried out for comparison. Results obtained with electromagnetic induction heating showed that this methodology performed largely better both in terms of time process and extraction yields.

## 1. Introduction

**Figure 1:**
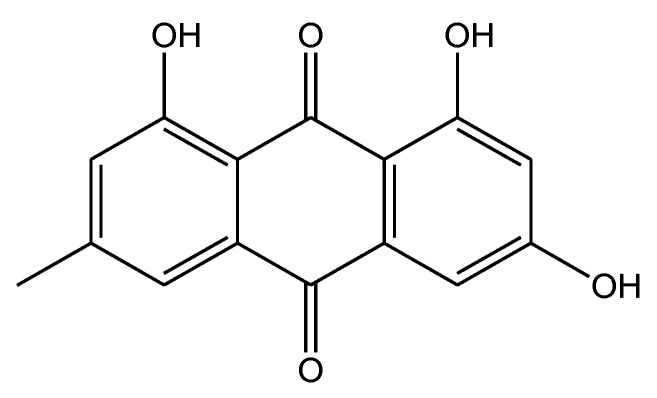
Structure of emodin.

Conventional extraction methodologies of plants include maceration, heating, or refluxing in the presence of solvents with different polarities [1]. Drawbacks currently associated with such processes often include the loss of the desired secondary metabolites due to the occurrence of several side reactions like hydrolysis, air oxidation, and chemical degradation in general. These are also due to the long operation time required to accomplish the above-mentioned experimental steps. Thus, quite recently, the attention of academic and industrial research has been focused on the search for alternative and more efficient plant extraction processes and these include ultrasound [2] and microwave-assisted techniques [3], pulsed electric field extraction [4], the use of supercritical fluids [5], and pressurized liquids [6]. However, most of the recent set-up methodologies may have some drawbacks in some cases (e.g., use of expensive equipment and maintenance). Hence, the search for novel, alternative, more economic, and easy to handle procedures to accomplish plant extraction is a field of current and growing interest. In this short communication, we wish to describe a simple, very rapid, and effective method based on the use of electromagnetic induction heating. Extraction and subsequent HPLC quantification of emodin (Figure 1) from the rhizome of Chinese rhubarb, *Rheum palmatum* L. (Polygonaceae) was selected as the reference process.

## 2. Results and Discussion

**Figure 2:**
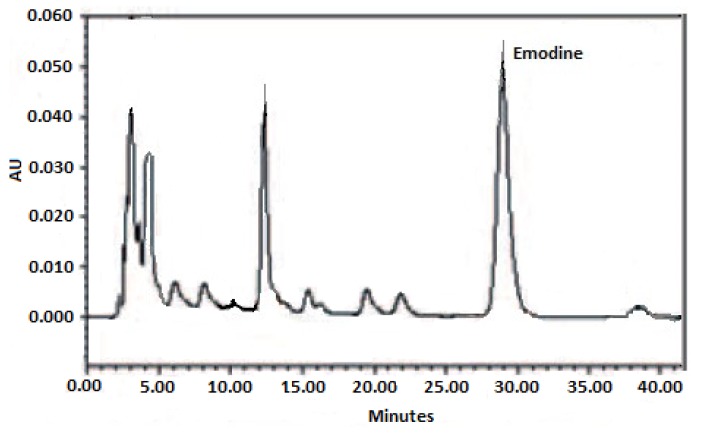
Chromatogram of the extract obtained from the rhizome of *R. palmatum* after electromagnetic induction heating application (AU = Absorbance Unit).

To test the effectiveness of electromagnetic induction heating as an efficient plant extraction methodology, four other processes with an equal amount of solvent and vegetable material were carried out: “classic” maceration (for 15 days), macerating at reflux temperature (85 °C for 4 h), ultrasound-assisted and microwave-assisted processes (80 °C both for 10 min), employing the same optimized conditions that we have adopted for the extraction of anthraquinones from plants belonging to the genus *Rhamnus* [7]. The indicated times and temperatures for these latter experimental steps correspond to the best extractive obtainable yields in the reference compound emodin. For the electromagnetic induction heating-assisted extraction of the rhizome powder of *R. palmatum*, the following parameters were screened: induction power (from 1 to 9, from 200 W to 1800 W) and time (10 s to 1 min). After several attempts, the optimized experimental conditions were set at an induction power of 9 (corresponding to 1800 W and 83 °C into the reaction vessel) and 30 s. The first value allowed a rapid boiling of the extraction solvent (<10 s), while extending the process time over 30 s. It did not result in better yields of emodin, and, more disadvantageously, a deep darkening of the extraction solution (probably indicating a chemical degradation of the chemical components) and a massive evaporation of the solvent (thus, rendering the subsequent work-up more difficult) occurred. The content of emodin in each of the five extracts obtained was assessed by HPLC analysis coupled to UV/Vis detection, following the well-validated methodology we set-up in 2010 for the anthraquinones screening in plants belonging to the genus *Rhamnus* [7]. The calibration curve of pure emodin was drawn using weighted (1/x^2^) linear least-squares regression analysis and shown to be linear along the entire concentration range applied, providing r^2^ values ≥ 0.9996. The retention times of emodin detected in each extract matched that recorded from chromatographic runs of pure emodin. Repeatability, precision, and trueness of the analytical methodology were assessed as previously reported [8]. Results of the quantification of emodin in extracts obtained by the different extraction methodologies listed herein are reported in Table 1, while Figure 2 illustrates the chromatogram of rhubarb extract obtained after application of electromagnetic induction heating. Figure 2 also accounts for the choice of emodin and *R. palmatum* as the reference compound. The best baseline separation (allowing a sharp quantification) of the peak of this anthraquinone and no co-elution of other phytochemicals were achieved among the several chemical standards and plant extracts that were screened for our purposes. Results from Table 1 clearly indicate how electromagnetic induction heating performed much better than all other extraction methods applied. The increase in emodin concentration was from nearly 3-fold to more than 5-fold. This positive trend may be explained by the most favorable features of electromagnetic induction heating in that a uniform heating across the whole of the extraction vessel can be achieved in a few seconds leading to a massive and rapid release of secondary metabolites from plant cells and preventing their chemical degradation, pending a short-lasting process being applied. Furthermore, this is particularly true in the case of anthraquinones from Chinese rhubarb, for which it has been demonstrated that heating represents an effective means to increase their extractive yields from rhizome [9].

To ensure that quantity recorded by HPLC analysis was the expression of the real value of free emodin originally contained in the plant and not contaminated by the addition of emodin as a result of hydrolytic processes of glycosylated forms that may occur during extraction processes, we exposed commercially available pure emodin-8-glucoside to the same experimental conditions employed to accomplish rhubarb extractions. Free emodin was then quantified by HPLC analysis using the same mobile phase and chromatographic run time. Values of the recovery of this anthraquinone are reported in Table 2.

It has been reported that several anthraquinones glycosides are thermally (100 °C or more, for times of 10 min or more) unstable during extraction processes, especially when MeOH/H_2_O mixtures with high percentages of this latter are used as extractive solvents [10]. In our case, a slight degree of hydrolysis was also recorded, but this was pronounced only in the case of maceration at reflux for 4 h. In all other trials, the degradation of emodin-8-glucoside was minimal (<2.4%). This may be due to the experimental conditions adopted that are different from those reported in the literature, especially for what concerns the solvent (EtOH instead of MeOH), operating temperatures (always <85 °C) and times (10 min or largely less as in the case of induction heating).

Although the one described herein is not effectively the first example in the literature about the application of electromagnetic induction heating for plant extraction purposes, previous reports are limited to the isolation of gross material, like pectin [11] or to the enrichment of extract in polyphenols [12]. In both cases, prolonged times to accomplish the extraction process were used (30–120 min). Moreover, in both cases, extractive yields were strongly dependent on chemico-physical parameters like pH and the electrolyte concentration of the extractive solution. In our case, the most advantageous features of the method described are the use of easy-to-handle equipment and the very short operational times. Even if preliminary, the findings described are very promising and have a great potential for application to a larger panel of classes of naturally occurring compounds from a wide array of plant sources, as well as for suitable modification for application in the industrial scale.

## 3. Materials and Methods

### 3.1. Plant Material

Rhizomes of *R. palmatum* were purchased from a local market (Chieti, Abruzzo region, Italy). Plant material was identified by the authors and a voucher specimen (RP-001-18) has been stored in the repository of the laboratory of Chemistry of Natural Products of the Department of Pharmacy of the University “G. d’Annunzio” of Chieti-Pescara. The vegetable material has been finely triturated by an Ultraturrax apparatus (IKA^®^-Werke GmbH & Co. KG, Staufen, Germany), before accomplishing all kinds of extraction.

### 3.2. Chemistry

Emodin (HPLC standard grade, purity ≥ 99.0%) was purchased from Extrasynthese (Genay, Lyon, France). Ethanol (UHPLC grade), methanol (UHPLC grade), and formic acid were purchased from Carlo Erba Reagents (DasitGroup-Carlo Erba Reagenti, Milan, Italy). H_2_O (HPLC grade, >18.2 MΩ cm resistivity) was purified from a double pass through Elix 3 and Milli-Q Academic equipment (Millipore, Bedford, MA, USA). Stock solutions of standard emodin (1.0 mg/mL in MeOH) were kept in amber vials in a refrigerator at 4 °C before injection into the HPLC apparatus.

### 3.3. Extraction Procedures

Maceration at room temperature and heating, ultrasound-assisted, and microwave-assisted extraction have been performed as already reported [3,8,13]. The electromagnetic induction heating assisted process was accomplished using a domestic apparatus (model Tillreda, Ikea, Sweden) with 9 power heating levels. A stainless pot with lid (compatible for induction heating, h = 7 cm, ∅ = 15 cm) was used as a container for plant material. For all extraction steps 5 g of powder of the rhizome of Chinese rhubarb and 100 mL of a H_2_O/EtOH 7:3 mixture as the solvent was employed. At the end of each process the suspension was filtered, and the resulting solution evaporated to dryness under vacuum. The resulting raw solid was re-suspended in MeOH (10 mg of dry extract in 100 μL), centrifuged, and, finally, aliquots of 20 μL of this solution were injected into the HPLC apparatus.

### 3.4. HPLC conditions and Method Validation

An HPLC apparatus Waters 600 coupled to a 2996 photodiode array detector and Empower v.2 Software (Waters Spa, Milford, MA, USA) for data acquisition, a GraceSmart RP18 (4.6 × 150 mm, 5 μm; Grace, Deerfield, IL, USA) column thermostatted at 24 ± 1 °C were employed to carry out analyses. The reference wavelength for the qualitative and quantitative determination of emodin was 435 nm. The mobile phase, consisting of a mixture of H_2_O–MeOH 40:60, *v*/*v* + 1% formic acid, was degassed by a Degasser Phenomenex mod. DG-4400 (Torrance, CA, USA) and used following an isocratic elution mode. The validation of the HPLC methodology described herein was achieved following the rules and recommendations laid down by the “Guidance for Industry-Bioanalytical Method Validation” of the Food and Drug Administration (FDA, USA). Chemical standard solutions to draw calibration curves at concentrations of 1.0, 10.0, 20.0, 30.0, 40.0, 50.0, 60.0, 70.0, 80.0, 90.0, and 100.0 µg/mL, were daily obtained by dilution of the stock solution of pure emodin. Quality control QC) samples at three different concentration, namely QC_low_ = 10.0 µg/mL, QC_medium_ = 40.0 µg/mL, and QC_high_ = 90.0 µg/mL were employed to evaluate the HPLC run in terms of limit of quantitation (LOQ), intra-and inter-assay precision, and accuracy [13]. In particular, these latter parameters were recorded from the chromatographic runs in duplicate of 3 sample solutions of LOQ and QC at 3 different concentration values of emodin accomplished on the same day and for 5 consecutive days. Calibration curves (6 per day along a period of 5 consecutive days) were estimated by correlation coefficients, slopes, and intercepts. The limit of detection (LOD) was extrapolated from the calibration graphics according to what reported in the guidelines provided by IUPAC [14].

## Figures and Tables

**Table 1:** Quantification of emodin in extracts of the rhizome of Chinese rhubarb.

Extraction Method *	Emodin Content (mg/g of Dry Weight)
A	2.81 ± 0.05
B	3.46 ± 0.07
C	1.98 ± 0.12
D	2.01 ± 0.12
E	9.98 ± 0.12

* A = maceration r.t., B = maceration at reflux, C = ultrasounds, D = microwaves, E = electromagnetic induction heating.

**Table 2:** Quantification of the chemical degradation of emodin from emodin-8-glucoside exposed to the same experimental conditions as accomplished for rhubarb extraction

Extraction Method *	% Free Emodin Recorded
A	0.44 ± 0.03
B	6.12 ± 0.06
C	1.44 ± 0.03
D	2.37 ± 0.04
E	0.88 ± 0.05

* A = maceration r.t., B = maceration at reflux, C = ultrasounds, D = microwaves, E = electromagnetic induction heating.
